# Testing the validity of the broaden-and build theory of positive emotions: a network analytic approach

**DOI:** 10.3389/fpsyg.2024.1405272

**Published:** 2024-09-24

**Authors:** Leopold Helmut Otto Roth, Celine Bencker, Johanna Lorenz, Anton-Rupert Laireiter

**Affiliations:** ^1^Faculty of Psychology, Department of Occupational, Economic and Social Psychology, Motivation Psychology, University of Vienna, Vienna, Austria; ^2^Faculty of Psychology, Department of Clinical and Health Psychology, University of Vienna, Vienna, Austria; ^3^Department of Psychology, Paris Lodron University Salzburg (PLUS), Salzburg, Austria

**Keywords:** positive emotions, upward spiral, broaden-and-build theory, resources, resilience, positive mental health

## Abstract

**Introduction:**

The Broaden-and-Build Theory of positive emotions is one of the best known and applied theories in Positive Psychology. It argues that positive emotions initiate an upward movement by opening up the mind and broadening thoughts and thus represents a counter model to the vicious circle-models of clinical psychology. The number of studies directly testing this theory in all components is scarce, ambiguities in the model impede clear inference.

**Method:**

To draw a conclusive picture on within-model processes, we applied network modeling on the components of the theory across two studies (*N*_1_ = 312; *N*_2_ = 302).

**Results:**

In both studies, the positive relationship between positive emotions, resources and life outcomes is well-supported, yet the role of broadening, as an intermediary component within these is questioned.

**Discussion:**

As the broadening component consistently deviated from the model’s predictions and thus did not contribute to the model as expected in either study, the validity of the Broaden-and-Build Theory in its current conceptualization is challenged, and our results point to the need to reassess the role of broadening.

## Introduction

It is the first sunny day after a week of rain and gray skies and we head out of the office to take a little break and soak up some sun. Just when we are almost out, our phone notifies us of an email. The headline already indicates that it is the good news we have been hoping for and we leave the building smiling and with a lighthearted mind. It is that moment when we see the trees blossoming that we usually just rush by. We suddenly hear birds singing, while we usually already have our heads at the desk before even entering the office. And just in this moment, we remember this interesting exhibition we heard about and had no time to visit in the last few months. Full of inspiration and with a cup of coffee in our hands, which just seems to smell better than usual, we get back to our work and somehow everything goes lighter this afternoon. We chat with colleagues and laugh together, and they seem more drawn to the conversation than when we are stressed and feel strained. After working off the last emails of the day fast and easily, it is time to head home and spend a longer-than-expected evening with friends and family, setting us up for good memories the next morning.

What seems like the trivial story of a good day describes the foundation of one of the best-known models of Positive Psychology (Fredrickson, 1998): the Broaden-and-Build Theory (BBT) of positive emotions. This theory was one of the earliest attempts to bring the conglomerate of positive feelings, openness to the environment, positive social interactions, positive ideas about the future, optimistic thinking, resilience to negative emotions etc. into a systematic theoretical concept beyond “simple” well-being. At its core, it describes four components that make up the functions of positive emotions (Fredrickson, 1998). (1) *Positive emotions* cheer up our mind and thus broaden our attention toward our environment and possible actions we could take. This cheered-up and (2) broadened mind (*broadening* component) helps us to (3) *build* resources over time, just like social connections, optimism, or resilience. Consequently, these resources promote further (4) *positive outcomes*, such as satisfaction with life, flourishing and, as an ultimate consequence, positive mental health. To close the loop, these resources and positive outcomes facilitate in return positive emotions and therefore create some sort of a (positive) spiral between the components, called an “upward spiral.” In addition, and at the same time, this upward spiral counteracts processes and spirals of negative emotions by their dissolution.

This idea was initially set up as a counterpart to the downward spiral or vicious cycle of negative emotions, anxiety, and depression, well known from clinical research (Garland et al., 2010). For the longest, in psychological research, positive emotions and their functions were unknown and rather neglected and thus not subject of research. It can be considered Fredrickson’s (1998) merit having established positive emotions as a field of research in psychology by putting their quality and functions into focus.

### Positive emotions

According to Fredrickson (1998), positive emotions can be defined from two perspectives: (1) their subjective quality as containing some kind of positive experience and (2) their functions by enhancing well-being and initiating the described upward spiral. Accumulating research on positive emotions since then supports their functional role in various domains, such as in the clinical field (e.g., Lambert et al., 2012; Lamers et al., 2012; Sin and Lyubomirsky, 2009), in pain management (Hanssen et al., 2017; Thong et al., 2017), social connections (Brown and Fredrickson, 2021), physical health, and health behavior (Fredrickson et al., 2021; Pressman et al., 2019). More specifically, singular positive emotions, such as gratitude, happiness, or hope (Chang E. C. et al., 2022; Chang O. D. et al., 2022; Cohn et al., 2009; Xiang and Yuan, 2021), have been explored for their unique effects, supported by the notion that they do not necessarily all account for the same latent construct (Roth and Laireiter, 2021).

### Research on the BBT

A diverse body of research on the BBT emerged throughout the past decades, mainly in terms of applying the theory in diverse fields of research and less about testing the model *per se*, which was done mainly by Fredrickson and her group (e.g., Fredrickson, 2004, 2013; Garland et al., 2010). Indeed, the model offers a well-applicable framework on how positive emotions, personal and social resources, cognition, and outcomes could interact either in each moment or across time. The following summary of recent research on the theory is not exhaustive but rather gives a selected overview of current findings and approaches.

In their study, Xiang and Yuan (2021) sought to examine the predictive effect of gratitude on satisfaction with life, with mindfulness and both benign and malicious envy as potential mediating factors. The theoretical framework posits that gratitude may have a broadening effect and potentially influence envy-related processes. Similarly, Johnson et al. (2021) investigated the relationship between mindfulness, positive emotions, resilience, and perceived stress. Testing multiple models against each other, non-judging and non-reacting were identified to be directly and indirectly related to resilience and perceived stress and emotions. Using structural equation modeling (SEM), Denovan and Macaskill (2017) investigated the relationship between resilience, leisure coping, and positive affect and their impact on stress and flourishing. The study showed that coping and resilience directly influenced flourishing but not perceived stress. Many similar and additional studies were conducted during the recent years, investigating the pathways between typical variables from positive psychology to test parts of the BBT in various contexts (e.g., Chang E. C. et al., 2022; Chang O. D. et al., 2022; Chen et al., 2021; Enete et al., 2022; Johnson et al., 2021).

Although many studies have been conducted in this field, three major limitations remain: (1) Definition and operationalization: Due to the ambiguity of the model’s definition, the broadening term is not clearly conceptualized, which hampers its operationalization. (2) Design and methods: Often, cross-sectional data are tested improperly with statistical models that assume a causal structure of the data, like structural equation modeling, regression analyses, or mediation models. (3) Partiality: Many studies have only examined specific facets of the theory, such as the broadening or building effects, neglecting to investigate their temporal sequence, functions, and reciprocal relationships with positive emotions, satisfaction with life, and mental health.

The present studies seek to overcome some of these limitations and deliver first insights into the model’s general mechanisms without assuming a causal structure to the data and without having collected longitudinal data. Using network modeling, the connections between the variables can be tested while controlling for the influence of the remaining ones in the network. While this cannot substitute for a thoughtful longitudinal assessment, the presented studies can provide preliminary guidance for a future re-conceptualization of the theory.

### Network modeling and network analysis

Recent theorizing and methodological advances allow researchers to adopt a new approach for analyzing multivariate and interdependent data through network modeling. This approach treats systems of variables as interconnected networks (Fried and Robinaugh, 2020), which can be investigated by a class of statistical applications, so-called *network models* (Boorsboom et al., 2022). The major advances and primary applications of these models are within the domain of psychopathology, where relationships between symptoms can now be modeled in more detail (Boschloo et al., 2015; Fried et al., 2015; Isvoranu et al., 2017). Unlike network models in other scientific branches, psychological networks are defined by variables and their relations with each other, rather than specific entities (Deserno et al., 2022). Models of this kind are called “Pairwise Markov Random Fields” (PMRF; Epskamp et al., 2022), where variables are referred to as *nodes*, which are connected through *edges* conveying information about the strength and direction of the relationship. If a relationship between two variables is depicted in a network model, these two variables are conditionally dependent. While dependence can be termed in many ways, the definition of independence is straight forward. Two variables (e.g., X1 and X2) are considered independent if knowledge of one does not provide information about the other. If this independence holds after controlling for a third variable (e.g., Y), or more, the variables are considered conditionally independent (X1 ⫫ X2 | Y; Waldorp et al., 2022). The relevance of a particular node in the network is quantified by measures of centrality, such as strength, betweenness, or closeness (Opsahl et al., 2010; Robinaugh et al., 2016). Most research is aiming beyond merely drawing a network graph but rather to conclude on an optimal model, given the data. This model ranges somewhere between a fully independent, with no connections between any nodes, and a saturated model, including every possible edge (Blanken et al., 2022). For continuous datasets, Gaussian Graphical models (GGM) and partial correlations are commonly used to compute the core model. The partial correlations refer to the edge between two nodes after conditioning on all other nodes in the network. To identify the optimal model, which is parsimonious without sacrificing performance, different algorithms can be applied, such as *thresholding*, *pruning*, *model search,* and *regularization* (Isvoranu and Epskamp, 2021). Regularization, an application penalizing complexity in statistical models, is borrowed from machine learning and applied in this article due to its generalizable network structures (Blanken et al., 2022; Hastie et al., 2001). Regularization can use different approaches; for multivariate estimation in GGMs, the graphical Least Absolute Shrinkage and Selection Operator (LASSO) is recommended (Friedman et al., 2008). LASSO creates sparse networks through setting edge weights to zero (Tibshirani, 1996), contrary to other methods, which shrink parameters. This procedure involves the tuning parameter λ which is hence directed through the Extended Bayesian Information Criterion (EBIC, Chen and Chen, 2008), which is specifically suitable for PMRFs (van Borkulo et al., 2014). The rigor of the EBIC is controlled through the manually set hyperparameter γ (set to 0.5 in this article). After estimating the model, its stability can be assessed through non-parametric bootstrapping (Fried et al., 2022; Hastie et al., 2015). Therefore, the computed bootstrapped confidence intervals cannot be used for significance testing in regularized models, which control for error variance already. The stability of centrality estimates can be computed through case-drop bootstrapping (Epskamp et al., 2018). Lastly, different groups can be compared for network equivalence, using network comparison tests (van Borkulo et al., 2023). These involve a re-estimation of networks by group, a permutation procedure which constructs the respective test statistic and finally the test of significance against the distribution of the statistic. In the present paper, tests for network invariance and global strength are applied. In case of specific hypotheses, targeted edges can be compared between groups as well (Fried et al., 2022).

### Current study

In two studies (*N*_total_ = 614), we explored the relation between commonly used components of BBT, including positive emotions, current thought-action-potentials (broadening), resources (building), and respective outcomes (e.g., satisfaction with life). To our knowledge, this approach is new to the field and is expected to deliver the most complete picture of dynamics within the BBT. While the BBT is a model with temporal components, we are yet confident that a cross-sectional network approach should be sufficient to take the equivalent of a snapshot of the between variable relationships. While we purposely avoid causal claims, we argue that due to the recursive nature of the hypothesized model, we can get useful and informative insights, even by cross-sectional data, as in our case, which can inform both theory and future studies.

## Study 1

### Approach

To test the relation between all components of the BBT, six core measures were identified from the literature and selected as indicators of positive emotions. To buffer against measurement dependencies, two measures for broadening and building were selected. After data collection and cleaning, a regularized network model, using multivariate estimation was computed between all variables. The analysis aimed to test the hypothesized directional links between positive emotions, broadening, building, and positive outcomes, with a particular focus on the expected strong association between positive emotions’ broadening function and positive outcomes. Consistent with the theoretical framework, positive emotions were expected to have weaker direct connections with building variables, while building measures were anticipated to have a positive association with both broadening indicators and positive outcomes.

### Methods

#### Sample

We collected *N* = 312 complete cases (German speakers), in late 2018/early 2019. Between female and male participants, balanced sampling was approximated (*n*_female_ = 194, *n*_male_ = 116, *n*_divers_ = 2). Age ranged from 18 to 79 years (*M* = 29.105, *SD* = 10.103, *Mdn* = 26). The minimum age for participation was 18 years. No other eligibility criteria were applied.

#### Procedure

The study was conducted as a cross-sectional online survey, spread through diverse social media. No incentive was offered for participation. Individuals, following the link to the study have been informed about the approximate length and the option to always withdraw from agreeing to participate in the study. After consent to participate, the described scales were presented in random rotation (except for the demographics at the end), to avoid cueing biases.

#### Measures

We used instruments used by Fredrickson and her colleagues in their publications, to enhance comparability of model outcomes and better assessment regarding measurement dependency. Emotions: as a measure of current positive emotions, we used the same measures as Fredrickson and Losada (2005) did and added serenity and inspiration to cover the 10 key positive emotions defined by Fredrickson (2013). The emotions were: amusement, awe, compassion, contentment, gratitude, hope, interest, joy, love, pride, sexual desire, serenity, and inspiration. As in the study of Fredrickson and Losada (2005), participants were asked to report the experienced intensity of each emotion within the past 48 h. Broadening: the broadening component of BBT is usually measured by instruments for action potentials and cognitive coping (Fredrickson and Branigan, 2005; Fredrickson and Joiner, 2002). In this study, the thought-action potential was measured by resembling the procedure of Fredrickson and Branigan (2005) using an open-ended version of the “*Twenty-Statements Test*” (TST) by Kuhn and McPartland (1954) on preferred actions. As in the original study, participants were asked to write down as many things they would like to do in the given moment. The number of activities was computed as a degree of broad mindedness. Further, the subscale “*Cognitive Analysis*,” taken from Moos’ *Coping Response Inventory* (*CRI*, Moos and Moos, 1988) was applied as the second *broadening* measure, just as in the study of Fredrickson and Joiner (2002) interpreting it as “*broad-minded coping*.” Building: to measure resources, we used scales for assessing optimism (Fredrickson et al., 2008) and resilience (Fredrickson et al., 2003). For optimism, we applied the three optimism items from the German version of the revised *Life Orientation Test* by Scheier and Carver (1985) (*LOT-R*, Glaesmer et al., 2008). Resilience was measured with the 13-item German resilience scale (*RS-13*) by Leppert et al. (2008). Outcome: the S*atisfaction with Life Scale* (SWLS) (Diener et al., 1985) was used, as for instance in Cohn et al. (2009). In this study, the German version of this scale by Glaesmer et al. (2011) was applied.

All analyses were performed in R (R Core Team, 2020).

#### Network analysis

To compute the model, we fitted a LASSO-regularized partial correlation network on the data. This approach computes all partial correlations between the variables and shrinks the weakest paths to zero (Epskamp et al., 2018). In this way, the model is cleared from statistical noise and only reports the most important connections. Two further benefits are accommodated in this approach. First, the partial correlations between two nodes present their connection after conditioning on all other nodes in the data set. Second, after regularization, the remaining paths do not need tests for significance, as they are meaningful to the model by the definition of LASSO-regularization. The result is therefore much purer than a typical correlation coefficient. Beyond the overall model computation, centrality indices are computed by variables. Following, the stability of the network solution was computed to quantify how robust the findings are. Using bootstrapping (*n* = 1,000), the robustness of the paths and of the centrality indices were computed. Last, using network comparison tests (NCT), demographic sub-groups were compared to evaluate whether specific groups would show diverging networks. This was quantified through network invariance (*M*) and global strength (*S*). Both global values were tested with the conventional alpha level (0.05; van Borkulo, 2018).

### Results

Descriptive statistics for all measures are presented in Table 1. In most cases instruments showed satisfying reliability. Only for cognitive coping (CRI), Cronbach’s alpha was below 0.80. The data can be found in the section of supplementary material as well as further fit indices.

**Table 1:** Descriptive results, study 1.

	*M*	*SD*	*Md*	*min.*	*max.*	*α*	*ꞷ*
PE	3.49	0.67	3.54	1.00	5.00	0.86	0.89
CRI	3.11	0.50	3.08	1.00	4.00	0.62	0.75
TST	3.63	2.75	3.00	0.00	15.00	---	---
LOT-R	3.60	0.91	3.67	1.00	5.00	0.82	0.84
RS-13	5.25	0.80	5.31	2.92	7.00	0.83	0.87
SWLS	4.98	1.23	5.20	1.00	7.00	0.85	0.89

PE, Positive emotions; CRI, Cognitive analysis scale from coping response inventory; TST, Twenty statements test; LOT-R, Optimism-subscale of the revised life-orientation test; RS-13, Resilience scale; SWLS, Satisfaction with life scale; Md, Median; α, Cronbach’s alpha; *ꞷ*, Total omega.

#### Network

**Figure 1:**
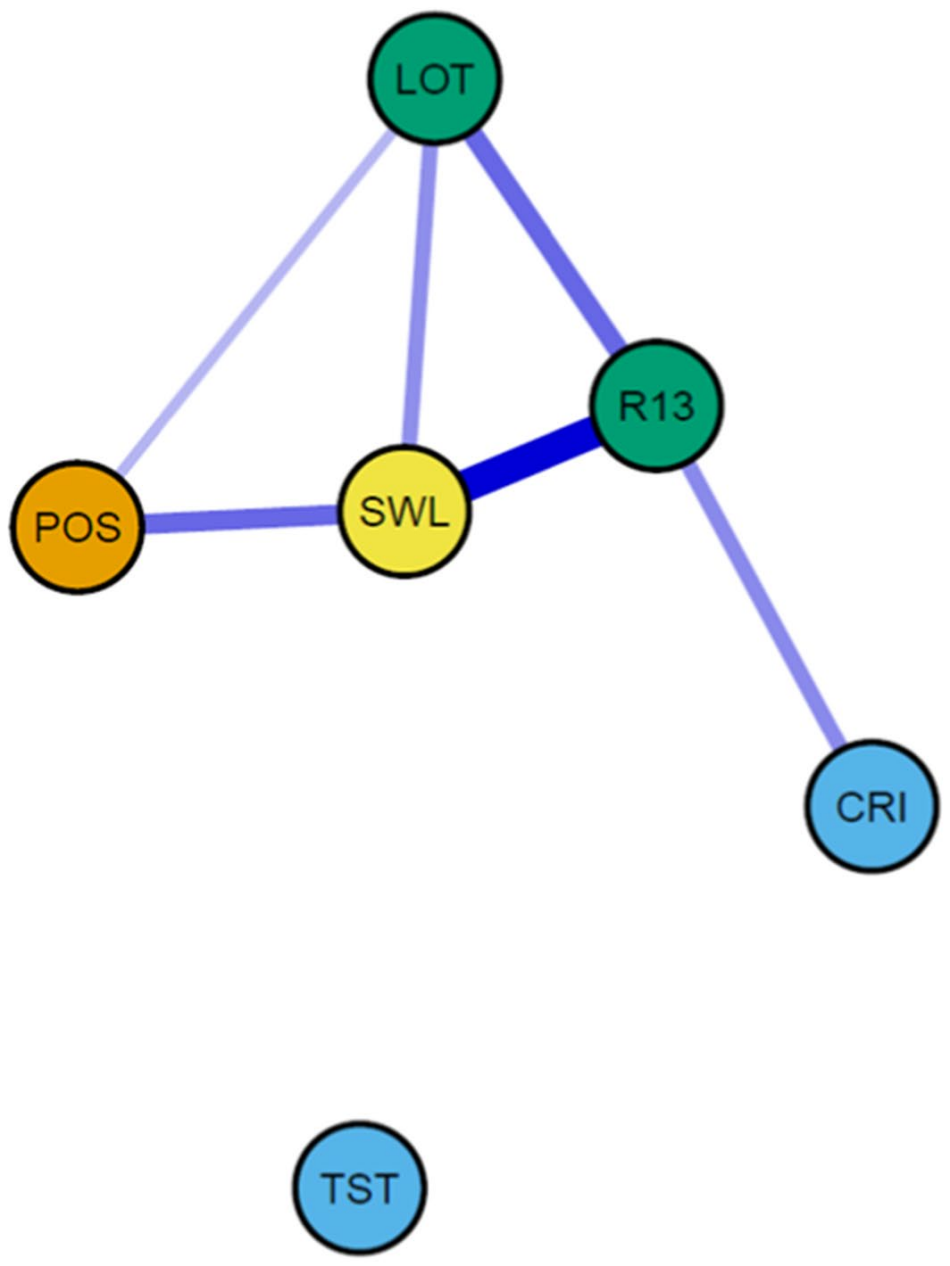
Network model, study 1.

**Figure 2:**
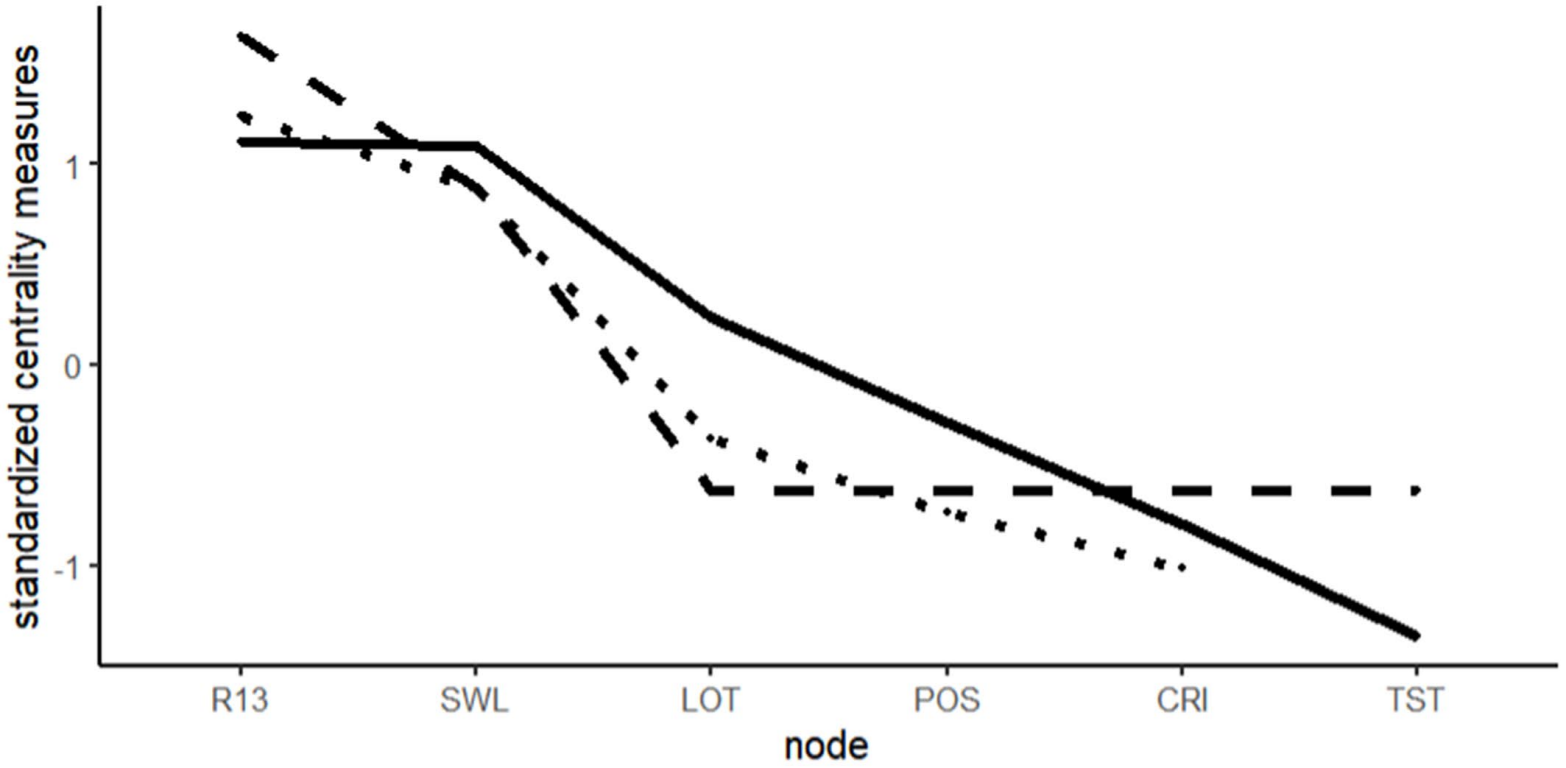
Centrality plot, study 1. Solid = strength, dashed = betweenness, and dotted = closeness.

The fitted network (threshold, EBIC = 0.50), based on LASSO-regularized partial correlations included six nodes (variables). Out of 15 possible edges, six remained in the final model (Figure 1). The graph shows that three parts of the model are well connected (positive emotions, building, and the outcome variable satisfaction with life). Resilience, satisfaction with life, and optimism are the most central variables in this network (Figure 2), with positive emotions following. Note that no result in closeness was computed for the TST as no parameter to other nodes of the edges remained in the model. All edges are positive, indicated by the blue lines.

The component of broadening appears to be quite isolated. One of the broadening components (*TST*, broadening the mind) does not show any meaningful edge with any other model component. *CRI* (broad-minded coping) only is related to resilience but does not appear to be meaningfully related to positive emotions, being however predicted by the theory. Based on these results, broadening variables do not seem to constitute a central component of the model.

#### Network stability

To examine the robustness of the results, networks can be evaluated through different bootstrapping procedures. The edges of the network can be tested through non-parametric bootstrapping, constructing confidence intervals around them. The further the interval is from zero, the more stable the parameter should be. Centrality measures are tested through case-drop bootstrapping. The resulting index *CS* quantifies how much of the sample can be dropped while retaining a correlation of 0.70 with the original results. The edges, included in the present model, appear stable after non-parametric bootstrapping (*n* = 1,000) except from the relation of positive emotions and optimism as well as resilience and broad-minded coping. While the stability for betweenness was weak *CS* = 0.051 and no case drop coefficient (*CS*) was computed for closeness, due to the missing edges with *TST*, strength was very stable (*CS* > 0.75). The respective plots can be found in the online material.

#### Network comparison test

By means of NCTs, different groups were compared with each other. Due to low case numbers, only very global group comparisons could be made (men vs. women; singles vs. in a monogamous relationship, and active vs. inactive sex life). There were no significant differences in either network invariance (*M* = 0.167, *p* = 0.881) nor global strength (*S* = 0.011, *p* = 0.962). The same was true for relationship status, comparing individuals being single vs. in a monogamous relationship (*M* = 0.258, *p* = 0.474, *S* = 0.149, *p* = 0.579) and no differences in networks were found between individuals with recent sexual activity or not (*M* = 0.217, *p* = 0.612, *S* = 0.447, *p* = 0.058). This allows us to conclude on similar network topologies and strengths between the tested populations, supporting certain generalizability of our findings.

### Summary of study 1

Set out to explore the cross-sectional interplay of relevant variables, used in *BBT*-research, study 1 delivered a series of interesting results. The original theory postulates a causal path from positive emotions to broadening, from there to building, preliminary terminating in positive outcomes and in reverse resulting in again enhanced positive emotions. Although the temporal sequence was not the subject of our specified design, we were still able to observe many of the components described in the theory. From the estimated network, the variables for positive emotions, resources and life satisfaction showed strong relations and appear to be connected in a robust manner. On the other hand, a serial or stepwise connection from positive emotions via broadening and building to satisfaction with life was not observed, as positive emotions and satisfaction with life mainly related directly to each other. Contrary to the theory, the broadening variables were either excluded (TST) or only loosely related to the rest of the variables. *Cognitive coping* was related to resilience but was not connected to positive emotions. It seems noteworthy that this pattern would have been unlikely to be found in a non-regularized manner, which optimizes the variables toward partial correlations and elimination of non-meaningful edges.

Study 2 was planned and conducted to replicate the findings and to test alternative measures for the broadening variable to explore the measurement dependency of the findings of study 1.

## Study 2

### Methods

#### Sample

We collected a sample of *N* = 302 complete cases (German speakers). Age ranged between 18 and 89 years (*M* = 33.27, *SD* = 14.72, *Md.* = 27). A similar gender distribution was found, as in study 1 (*n*_female_ = 181, *n*_male_ = 120, *n*_diverse_ = 1). The acquisition followed the same procedure as in study 1 and took place in early 2020 (before the outbreak of COVID-19 in Europe).

#### Procedure

The same procedure as in study 1 was applied, 1 year after the first data collection.

#### Measures

For optimism, resilience and life satisfaction, the same measures as in study 1 were applied. Positive emotions: The mDES (Fredrickson, 2013) was used in its German version (Brandenburg and Backhaus, 2015). For the current analysis, only the 10 positive emotions were considered. The items were rated on a five-point scale (0 = not at all; 4 = extreme) with respect to the experience within the last 48 h. Results were averaged for analysis. Broadening: we changed the respective measures in this model component, to provide a broader perspective in combination with study 1. First, we translated the *Range and Differentiation of Emotional Experience Scale* (*RDEES*, Kang and Shaver, 2004) to German. This scale is designed to measure the breadth of emotional experience as well as the degree of differentiation between emotions. Following the original instrument, items have been rated on a seven-point scale (1 = not at all characteristic for me; 7 = extremely characteristic for me). Further, the *German Brief COPE* (Knoll et al., 2005) was used to assess active-functional and cognitive-functional coping. The scale consists of 28 items in total, using a four-point Likert scale (1 = not at all; 4 = very much).

### Results

#### Descriptives

Descriptive statistics of all measures are displayed in Table 2. As in study 1, most instruments showed satisfying reliability. For cognitive coping (COG) and, additionally, for LOT-R, Cronbach’s alphas were below 0.80. The data can be found in the Supplementary material as well as further fit indices.

**Table 2:** Descriptive results, study 2.

	*M*	*SD*	*Md*	*min.*	*max.*	*α*	*ꞷ*
PE	3.12	0.67	3.20	1.20	4.70	0.86	0.89
COG	2.22	0.43	2.21	1.00	3.58	0.67	0.74
AFC	2.67	0.57	2.75	1.12	4.00	0.79	0.87
Diff	4.71	0.94	4.79	2.00	6.93	0.88	0.92
LOT-R	3.69	0.85	3.67	1.67	5.00	0.71	0.72
RS-13	5.35	0.80	5.38	2.00	7.00	0.87	0.91
SWLS	5.11	1.09	5.40	1.40	7.00	0.90	0.96

PE, Positive emotions; COG, Cognitive functional coping of the Brief Cope; AFC, Active-functional coping of the Brief Cope; Diff, Range and differentiation of emotional experience scale; LOT-R, Optimism-subscale of the revised life-orientation test; RS-13, Resilience scale; SWLS, Satisfaction with life scale; Md, Median; α, Cronbach’s alpha; *ꞷ*, Total omega.

The identified network from study 2 revealed strong similarities to study 1. The relationship between positive emotions, building measures, and life satisfaction appears essentially unchanged. The role of the broadening measures appears inconclusive again. While differentiation is isolated from the remaining network with no connection to any of the variables, cognitive coping is only related to positive emotions through active-functional coping and does not directly contribute to the network. Differentiation is only related with cognitive coping, which hence is only positively related with active-functional coping as well as negatively related to resilience. Active-functional coping is directly related to positive emotions, optimism and resilience but does not appear to fulfill the hypothesized role of connecting the variables in a highly central manner. That said it appears as if the measures for broadening do influence the network, yet not as predicted by the original theory.

As in study 1, the centrality measures illustrate that strength was mostly distributed between the non-broadening variables, apart from active-functional coping, which are listed on the lower end of the scale. This supports the notion that their role within the variables system is rather secondary.

#### Network stability

**Figure 3:**
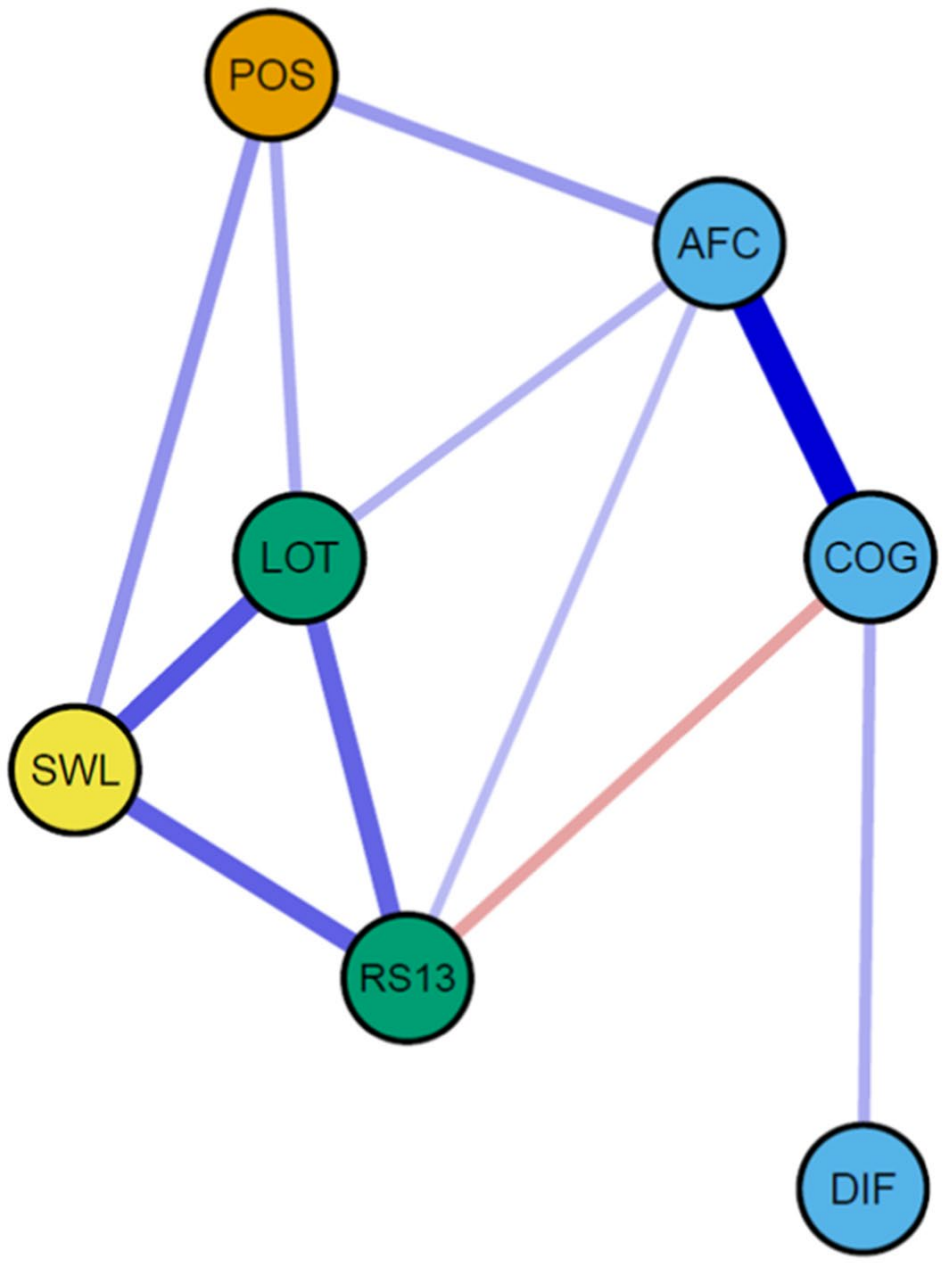
Network model, study 2.

**Figure 4:**
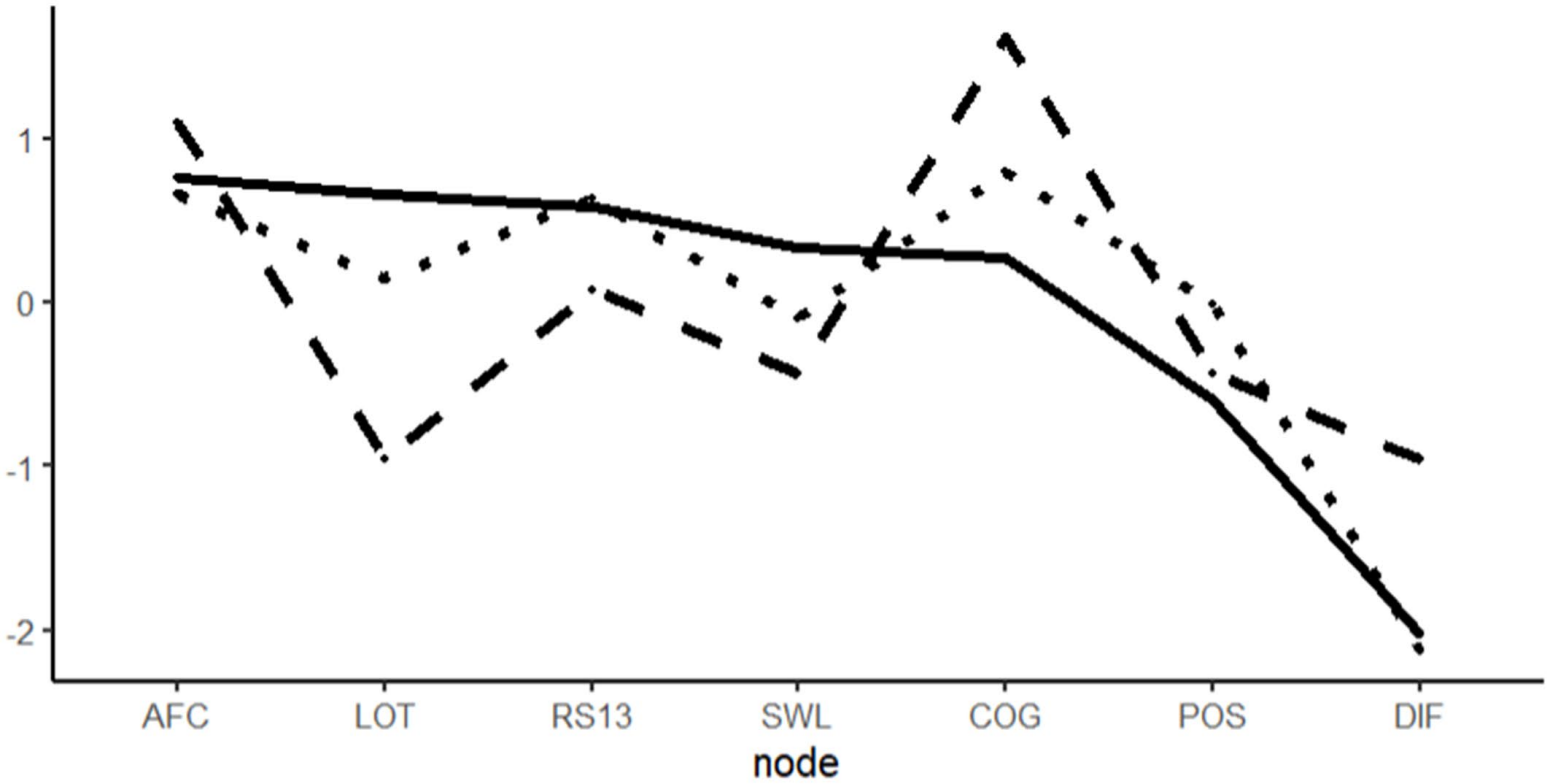
Centrality plot, study 2. Solid = strength, dashed = betweenness, and dotted = closeness.

Bootstrapped edges, included in Figure 3, appear mostly stable apart from the edges connected to positive emotions. Due to the questionable role of the broadening measures, network stability indices were again weak (betweenness: 0.00; closeness: 0.05; strength: 0.44). The respective plots can be found in the Supplementary material (Figure 4).

#### Network comparison test

No network differences were found between female and male participants as well as between participants with and without children (Table 3). We observed significant network variance between employed and unemployed participants. The respective plots are to be found in the Supplementary material.

**Table 3:** NCT results, study 2.

	*M*	*p*	*S*	*p*
Gender	0.292	0.363	0.007	0.992
Employment status	0.582	0.002	0.635	0.437
Children	0.243	0.807	0.352	0.673

M, Network invariance; S, Global strength.

## Discussion

### Summary

Using network modeling and network analysis, we investigated the interplay of variables, commonly involved in the broaden-and-build theory of positive emotions. We found substantial support for a strong interplay between positive emotions, building measures, and positive life outcomes. The role of broadening measures used to capture the process of changed thought-action repertoires, however, remained questionable. In both studies, they did not seem to act as hypothesized by connecting the model parts in a meaningful way. Due to the loose connection of the broadening measures to the rest of the model, the overall model cannot be supported. An exploratory series of network comparisons from our two independent studies did not reveal meaningful differences in model composition except from employment status. It appears noteworthy at this point that we examined a model with distinct temporal components in a cross-sectional manner and therefore recommend interpreting the findings only in a preliminary manner.

### Implications for the broaden-and-build theory of positive emotions

The current study raises questions about the role of the broadening component within the framework of BBT. While the other components of the model seem to be well interrelated, broadening measures were found to be rather isolated from the remaining system. This may be due to the lack of a temporal order in our study. However, to our knowledge, the theory does not make any clear statement on the timing of the variables within the model. Given the hypothesis of a continuous upward spiral of the model, it appears plausible to expect that the interrelationship between the components should be observable in a cross-sectional approach. Therefore, as one central consequence, we assume that the model is well-functioning without the broadening component as well. Until the role and measures of broadening are not captured better in a conceptual and empirical sense, we can conclude that positive emotions, resources, and life satisfaction are well interrelated and meaningful for each other, also without the variable of broadening the mind as a necessary intermediate stage or phase. In our view, this conclusion remains valid even if we keep in mind that we applied a cross-sectional design in both studies.

### Network modeling in positive psychology?

As demonstrated in the current studies, network modeling holds potential for the field of positive psychology. The approach allows to reach beyond often limited insight into multivariate patterns without overly strong assumptions. As seen in the case of the BBT, directionality, and causality might be not straightforward. While precisely planned studies on specific effects clearly contribute to the understanding of fine-grained mechanics, mere hypothesis testing of specific emotions or specific traits cannot be the sole advance toward understanding complex systems of human functioning. To gain clearer perspectives on broader landscapes of measures, the network approach can offer a strong toolbox to observe complete models or complex relationships and facilitate the generation of future hypotheses.

### Limitations

Although, as argued earlier, from our perspective longitudinal data are not strictly necessary to study the BBT, the cross-sectional nature of our two studies limits the interpretability of the results we obtained. Since the model also makes causal assumptions about the temporal course of each variable, it seems reasonable and necessary to also use prospective longitudinal data to test these assumptions. Beyond that, the selection of the variables, while drawn from a specific literature, remains arbitrary. While optimism and resilience are well-established resources in positive psychology (Färber and Rosendahl, 2020; Heekerens and Eid, 2021; Shanahan et al., 2021), future studies are needed to test the generalizability of the observed patterns. Last, we observed the weakest reliabilities in measures of broadening, which might put the general robustness of these measures into question.

### Future directions and implications

Two central implications emerge from our results: The first relates to the conceptual level of the theory and the second to its empirical testing. At the conceptual level, necessary further development and sharpening of the BBT, especially with respect to its broadening component, is needed. At the moment, this component seems to be neither conceptually nor theoretically adequately conceived. One can assume that the phenomenon of broadening the mind may be too short-sighted and include other, perhaps more important theoretical components, such as cognitive or affective ones. These could be thought of and formulated as counterparts to the well-known worrying or rumination elements of vicious cycles (Moorey, 2010). At the same time, these could also play an important role in broadening and opening of the mind in the sense of the BBT.

Empirically, continuing research presented here in a careful causal manner, the sustained application of network models appears justified. This enables researchers to observe a complex system of variables in various ways and reduce the complexity to the most meaningful substrate. To gain further insight into the BBT and Positive Psychology from a network perspective, two directions appear specifically promising: (1) a longitudinal extension to monitor the development of the variables interplay across time; (2) large-scale samples on more variables could offer a better understanding and insight for future theorizing. Overall, however, research on BBT should also change its focus in the future. Applications should not be the primary instance of research, but first and foremost the theory which must be tested with greater rigor and developed further.

### Conclusion

The BBT elicited mixed evidence in our two studies. While many of the model’s components showed meaningful relations and were robust across demographic groups, broadening consistently deviated from the model’s predictions. Based on our findings, we discussed two major implications for future research, to further develop and refine BBT theoretically, as well as conducting longitudinal studies with bigger samples and considering a broader range of measures. All in all, the primary focus of research should lie on the advancement of the theory and less on further searches for new applications.

## Data Availability

The datasets presented in this study can be found in online repositories. The names of the repository/repositories and accession number(s) can be found at: https://osf.io/zwm4e/?view_only=c1ac3162592c4ba196dfa66da48b7b5c.
